# Pericardial methotrexate-induced lymphoproliferative disorder in a patient with rheumatoid arthritis

**DOI:** 10.1093/rap/rkaf065

**Published:** 2025-06-04

**Authors:** Miyu Wakatsuki, Hiroyuki Yamashita, Shintaro Aozaki, Takuya Harada, Yusuke Nakamichi, Hiroshi Kaneko

**Affiliations:** Division of Rheumatic Diseases, National Center for Global Health and Medicine, Shinjuku, Tokyo, Japan; Division of Rheumatic Diseases, National Center for Global Health and Medicine, Shinjuku, Tokyo, Japan; Division of Rheumatic Diseases, National Center for Global Health and Medicine, Shinjuku, Tokyo, Japan; Division of Rheumatic Diseases, National Center for Global Health and Medicine, Shinjuku, Tokyo, Japan; Division of Rheumatic Diseases, National Center for Global Health and Medicine, Shinjuku, Tokyo, Japan; Division of Rheumatic Diseases, National Center for Global Health and Medicine, Shinjuku, Tokyo, Japan

Key message

- Pericardial methotrexate-associated lymphoproliferative disorder can occur in patients with rheumatoid arthritis.

Dear Editor, Several studies have shown a significant association between RA and lymphoproliferative disorders (LPD) [1]. The incidence of LPD is 2.0–5.5 times higher among patients with RA than in the general population [2]. Patients with RA treated with MTX can develop LPD, which is known as MTX-associated LPD (MTX-LPD), due to immunosuppression and EBV reactivation [1]. Approximately 50% of MTX-LPD has been reported to undergo spontaneous regression after MTX cessation [1]. Extranodal lymphomas occur in approximately half of MTX-LPD cases. Cardiac lymphomas are rare, accounting for only 0.03% of all lymphomas [3], and the incidence of cardiac MTX-LPD is extremely low. Only three cases of cardiac MTX-LPD have been reported in the literature [3–5]. This report presents the first case of pericardial MTX-LPD.

A 69-year-old woman was hospitalized due to dry cough and dyspnea on exertion for 4 days. The patient was diagnosed with seropositive RA 13 years ago, started on MTX, and maintained remission on MTX 6 mg and etanercept (ETN) 50 mg/week. Upon admission, her vital signs were stable. Blood tests revealed elevated levels of lactate dehydrogenase (LDH) (696 U/l), C-reactive protein (5.19 mg/dl). Given the history, soluble interleukin-2 receptor (sIL-2R) was ordered at 888 U/ml (cutoff: 500 U/ml; sensitivity: 79%, specificity: 47% for malignant lymphoma) [6]. Chest radiography and CT revealed a pericardial effusion (Fig. 1A). On transthoracic echocardiography, a large pericardial effusion was noted, but no tamponade signs or cardiac mass lesions were observed. The haemorrhagic pericardial effusion (1000 ml) was removed over 3 days using an indwelling drain. Immunohistochemistry was performed on cell blocks prepared from the pericardial effusion, which revealed numerous large atypical lymphocytes with condensed chromatin and indented nuclei (H&E; Fig. 1B). The malignant cells showed immunoreactivity for CD20 (Fig. 1C) and CD79a, but no immunoreactivity for CD3, CD5, CD30, CD10, HHV-8, and EBER-ISH. These findings were consistent with the diagnosis of diffuse large B-cell lymphoma. Considering the patient’s history of MTX use, she was finally diagnosed with MTX-LPD, and MTX and ETN were discontinued. Meds were discontinued, symptoms abated, and no pericardial effusion re-accumulation was noted by day of discharge. At 2 weeks after MTX cessation, fluorine-18 fluorodeoxyglucose positron emission tomography/CT (FDG-PET/CT) showed no accumulation globally and further demonstrated absence of re-accumulation within the pericardium (Fig. 1D). Additionally, recovery of reduced lymphocyte count and marked reduction in sIL-2R and LDH levels were observed. Fig. 1E shows the course of the patient’s clinical and laboratory findings. The patient experienced flare-ups of arthralgia and was started on salazosulfapyridine on post-discharge day 6 and iguratimod on day 20. However, RA activity was not suppressed. Therefore, tocilizumab (TCZ) 162 mg/week was started on day 42, and RA activity gradually improved. On follow-up 7 months after discharge, RA remained in remission, and no signs of lymphoma regrowth, such as pericardial effusion re-accumulation or elevated LDH and sIL-2R levels, were observed.

**Figure 1.**
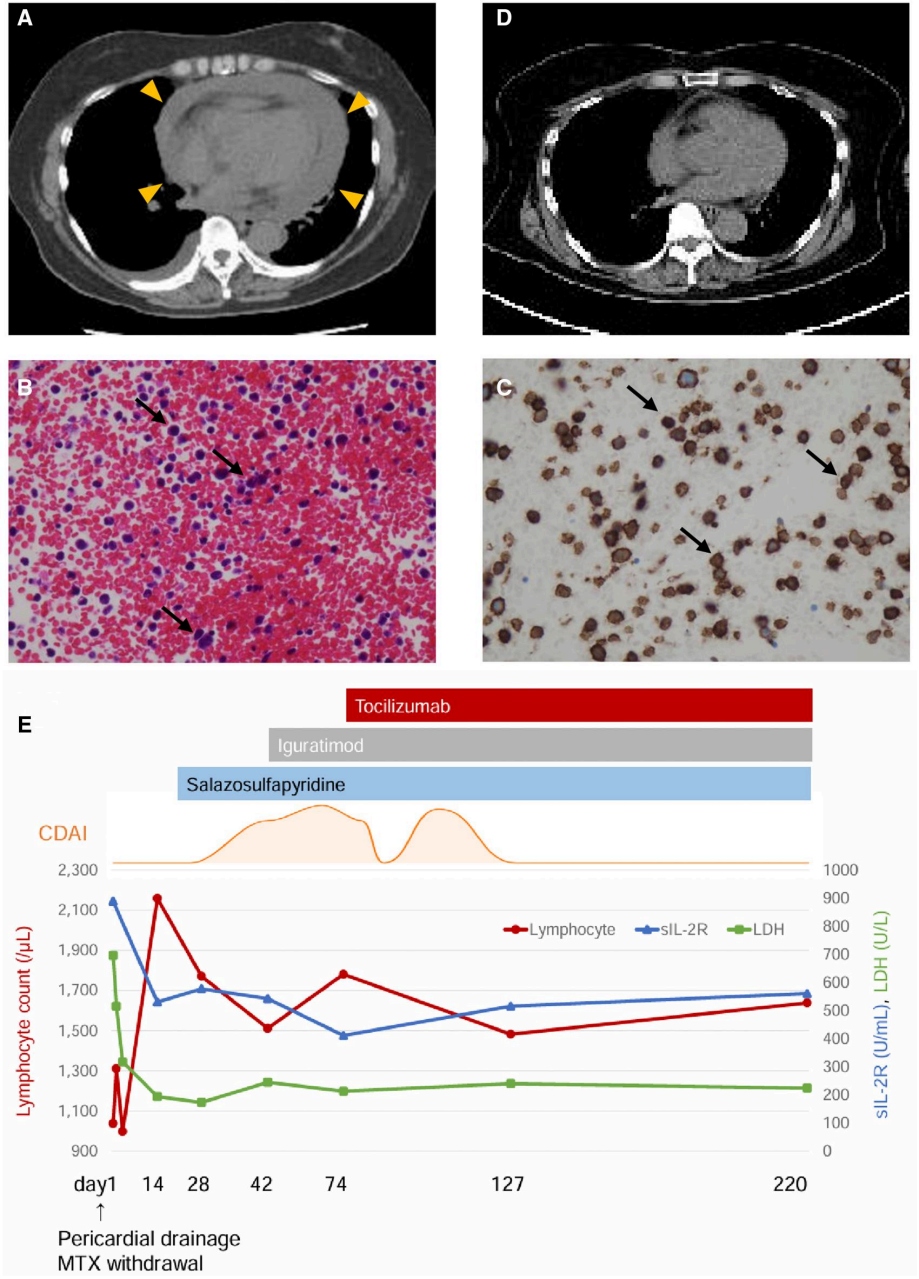
Imaging, histological findings, and the patient’s clinical course. (A) A CT image showing significant pericardial effusion. (B) Numerous large atypical lymphocytes with condensed chromatin and indented nuclei (H&E staining; original magnification ×400). (C) Immunohistochemical staining showing cytoplasmic CD20 positivity (original magnification ×400). (D) CT image 2 weeks after methotrexate withdrawal. (E) The patient’s clinical course. After admission, pericardial drainage was performed, and MTX was discontinued, leading to improved dyspnea. The patient was discharged on day 8. Recovery of the reduced lymphocyte count and a marked decrease in sIL-2R and LDH levels were observed. FDG-PET/CT performed on day 14 showed no accumulation and resolution of the pericardial effusion. Although follow-up FDG-PET/CT was not performed, periodic chest X-rays showed no signs of pericardial effusion re-accumulation. Flare-ups of arthralgia were managed effectively with salazosulfapyridine, iguratimod, and later tocilizumab. CDAI: Clinical Disease Activity Index; sIL-2R: soluble interleukin-2 receptor; LDH: lactate dehydrogenase

To the best of our knowledge, this is the first reported case of pericardial MTX-LPD. Unlike our case, in three previously reported cardiac MTX-LPD cases, MTX-LPD was not confined to the heart, and the cardiac lesions were mass lesions [3–5]. Although no mass lesions were observed in our case, the diagnosis was made by creating a cell block from the pericardial effusion. In all cases, including our case, the histological diagnosis was diffuse large B-cell lymphoma. Although cardiac non-Hodgkin’s lymphoma has been reported to be associated with a poor prognosis, with a median survival of 3 months [7], all reported cases of cardiac MTX-LPD spontaneously regressed after MTX discontinuation. Peripheral lymphocyte count recovery after MTX discontinuation is a characteristic of spontaneous regression of lymphoma [1], which was also observed in our case. In our case, FDG-PET/CT performed 2 weeks after MTX discontinuation showed no accumulation in any organs. A previous study showed MTX-LPD relapse in 19% of cases after spontaneous regression, with two-thirds of these relapses occurring within the first 2 years [8]. In our case, no signs of relapse were observed 7 months after MTX discontinuation. Continuous and careful monitoring during outpatient follow-up is necessary.

Following the discontinuation of MTX and ETN, the patient experienced arthritis flare-ups, prompting the initiation of TCZ therapy. After TCZ administration, RA activity was improved. A multicentre study involving 752 patients with RA-associated LPD showed that TCZ monotherapy was associated with a lower frequency of regrowth after spontaneous regression. Given that IL-6 is involved in diffuse large B-cell lymphoma pathogenesis through the signal transducer and activator of transcription three signalling pathway, TCZ regimens are a good option for treating RA after the onset of LPD [2].

In conclusion, MTX-LPD can occur in patients with RA, which manifests as a localized and primary pericardial effusion. Therefore, recognizing the possibility of MTX-LPD when pericardial effusion is detected in patients with RA undergoing MTX treatment is essential.

## Data Availability

Data are available from the corresponding author upon request.
